# Fabrication of magnetic niosomal platform for delivery of resveratrol: potential anticancer activity against human pancreatic cancer Capan-1 cell

**DOI:** 10.1186/s12935-024-03219-2

**Published:** 2024-01-29

**Authors:** Akram Firouzi Amandi, Zahra Bahmanyar, Mehdi Dadashpour, Mehrnoosh Lak, Mohammad Natami, Yusuf Döğüş, Mahsa Alem, Omid Ali Adeli

**Affiliations:** 1 Department of Medical Immunology, Facultyof Medicine, Tabriz University of Medical Sciences, Tabriz, Iran; 2 School of Pharmacy, Shiraz University of Medical Sciences, Shiraz, Iran; 3 Department of Medical Biotechnology, Faculty of Medicine, Semnan University of Medical Sciences, Semnan, Iran; 4 Cancer Research Center, Semnan University of Medical Sciences, Semnan, Iran; 5 School of Medicine, Shahid Beheshti University of Medical Sciences, Tehran, Iran; 6 Department of Urology, Shahid Mohammadi Hospital, Hormozgan University of Medical Sciences, Bandar Abbas, Iran; 7 Department of Medical Biochemistry, Faculty of Medicine, Cukurova University, Adana, Turkey; 8 Department of Microbiology, Faculty of Veterinary Medicine, Urmia University, Urmia, Iran; 9 Department of Pathology, Lorestan University of Medical Sciences, Khorramabad, Iran

**Keywords:** Resveratrol, Niosome, Magnetic nanoparticle, Cytotoxicity, Pancreatic cancer cell

## Abstract

Recently, the presence of different nanoparticles (NPs) has developed targeting drug delivery in treatment of cancer cell. Targeted drug delivery systems using NPs have shown great promise in improving the efficacy of intracellular uptake as well as local concentration of therapeutics with minimizing side effects. The current study planned to synthesized resveratrol-loaded magnetic niosomes nanoparticles (RSV-MNIONPs) and evaluate their cytotoxicity activity in pancreatic cancer cells. For this aim, magnetic nanoparticles (MNPs) were synthesized and loaded into niosomes (NIOs) by the thin film hydration technique and then characterized via DLS, FT-IR, TEM, SEM and VSM techniques. Moreover, the cytotoxic activity of the RSV-MNIONPs on the Capan-1 cells line was assessed by the MTT test. The distribution number of RSV-MNIONPs was gained about 80 nm and 95 nm with surface charge of − 14.0 mV by SEM and TEM analysis, respectively. RSV loading efficacy in NIOs was about 85%, and the drug releases pattern displayed a sustained discharge with a maximum amount about 35% and 40%, within 4 h in pH = 7.4 and pH = 5.8, respectively. The cytotoxicity of the RSV-MNIONPs in the presence of an external magnetic field is higher than that of the RSV, indicating enhanced cellular uptake in their encapsulated states. Furthermore, RSV loaded MNNPs were found to induce more cell cycle arrest at the G0/G1 checkpoint than free RSV. Compared with RSV-treated cells, the mRNA expression levels of BAX, Bcl2, FAS, P 53, Cyclin D and hTERT, were significantly changed in cells treated with RSV loaded MNNPs. The niosomes NPs approaches have been widely used to attain higher solubility, improved bioavailability, enhanced stability, and control delivery of RSV. Our formulation displayed antitumor activity and can be considered an appropriate carrier with a great potential for future usage in cancer therapy.

## Introduction

Commonly, tumor can be definite as an illness in which a group of unusual cells divides uncontrollably, dissimilar the regular cell division rules [1]. Many of people are being suffered from tumor, and the death affected by various types of tumor is intensely rising in the worldwide [2, 3]. Pancreatic cancer (PC) is one of the greatest disturbing of all malignancies and major digestive gland tumor [4]. Moreover, the cause of pancreatic cancer is multifactorial and complex, cigarette smoking and family history are dominant [5]. It has been described the fourth leading reason of cancer death in both males and females in the United States [6]. Even though development in medicinal knowledge that has enhanced the survival rate of numerous cancers, the survival rate of PC has remained dismal with a five-year survival frequency of only 9% [4]. The common types of treatment for cancer has advantages and drawbacks, and combination therapy is critical to acquire the best results [7]. In consequence, novel methods and objects necessity to be established. Recently, the research for novel drugs that are expected to have an effect against cancer types, and the interest in research on natural product has increased.

Resveratrol (3, 5, 4ʹ -trihydroxystilbene) (RSV), a natural polyphenol and phytoalexin, has drawn considerable attention in the past decade due to its wide variety of therapeutic activities such as anticancer, anti-inflammatory, and antioxidant properties [8]. In numerous kinds of cancer cells, RSV stimulate apoptosis of cancer cell. Nowadays, the antitumor property of RSV is well-known to be connected to the presence of flavonoid factors and some strategies to combat cancer cells, such as arresting of the cell cycle, hindering invasion, angiogenesis and migration via the many molecular events [9]. However, its poor water solubility, low chemical stability, and short biological half-life limit its clinical utility [10]. As a result of breakthroughs in nanotechnology and nanomedicine, development of novel approaches of drug delivery is essential. Recently, various drug delivery approaches such as polymer nanoparticles (NPs), nanofibers, metal, micelles, NIOs and liposomes have been developed [11–15].

Niosomes (NIOs) are bilayer nonionic surfactant agent delivery system that make them able of encapsulating hydrophobic drugs into the bilayer and hydrophilic drugs inside the core. Structurally, NIOs are similar to liposomes but have greater chemical stability which makes them superior to liposomes [16]. Targeted delivery of drugs to tumor sites is a crucial aspect of cancer treatment, and drug delivery systems, such as niosomes, play an important act in attaining this goal [17].

Magnetic drug delivery systems are an exciting area of research and hold promise for improving the precision and effectiveness of cancer treatment. Magnetic nanoparticles (MNPs) are a group of NPs that can be employed using magnetic fields [18, 19]. Also, MNPs have been broadly used in hyperthermia, target drug delivery system, imaging, and the controlled release of medicine and extraction of biomolecules [20]. Hopeful outcomes are described about stimulus-responsive drug release at the target tumor site applying an external magnetic field [21]. Within the last decades, studies have focused on magnetic delivery approaches for vesicular drug delivery system, nevertheless, MNIONPs have not been examined extensively.

During the present work, we designed an innovative approach to drug delivery, incorporating MNPs into niosomal formulations for targeted delivery of RSV to Capan-1 cell pancreatic cancer cells. To attain this, MNPs were synthesized and loaded into RSV-NIOs by the thin film hydration technique and then characterized in size, shape, drug entrapment efficiency, magnetic particles content, and in vitro drug release rate Furthermore, MTT assay and flow cytometry have been done to assess a possible cytotoxicity effects of MNIONPs on Capan-1 cell pancreatic cancer cells in the presence or absence of an external magnetic field.

## Materials and methods

### Materials

Human pancreatic cancer Capan-1 cell was purchased from the Institut Pasteur Cell bank, Iran and maintained according to recommendations. Resveratrol, Fetal bovine serum (FBS) and trypsin–EDTA, dimethyl sulfoxide (3,4,5-Dimethyl thiazol-2- yl)-2,5 diphenyl tetrazolium bromide (MTT), PVA (Polyvinyl alcohol) and cholesterol, non-ionic surfactants (Span-60), FeCl3, methanol and chloroform were supplied from Sigma Aldrich (St. Louis, MO, USA). Penicillin–Streptomycin, and RPMI-1640 were taken from Gibco BRL; The first Strand of cDNA synthesized with the Kit purchased from Fermentas (Vilnius, Lithuania) and Syber Green PCR Master Mix kit was provided from Roche (Germany).

### Synthesis of MNP

The synthesis of MNPs has been described previously [22]. In brief, a mixed solution of ferrous and ferric ions (molar ratio, 1:2) was prepared by dissolving 6.2 *g* FeCl_2_·4H_2_O and FeCl_3_·6H_2_O in 80 mL deionized water and solution was stirred vigorously under N2 atmosphere at room temperature for 10 min. Then, a formed black precipitate was collected with an external magnet, washed several times with ethanol and deionized water, and dried in vacuum at 60 ℃.

### Synthesis of RSV-MNIONPs by thin film hydration technique

The synthesis of MNPs loaded NIOs was based on the thin-film hydration method according to the protocol stated by Firouzi Amandi et al. [23]. Span 60, Tween 80, and cholesterol were fixed with (1:2:12 ratio), and chloroform and methanol solvents (with a 1:1 ratio) were then added to create the mixture. In a rotary evaporator, the organic phase was evaporated at 60 ℃ under reduced pressure to create a thin layer in the round bottom flask. After 2 h, the acquired solution was separated from the rotary and placed at room temperature for 60 min. PBS was used to rehydrate the thin film at a pH of 7.4.

The hydrated NIOs was then sonicated for 10 min at 85 percent amplitude, 135 W power, and 20 kHz frequency using a sonicator to create a uniform NIOs. Because of its lipophilic nature, RSV is added in the first stage of synthesis, and MNPs ferrofluid (nanoscale ferromagnetic or ferrimagnetic particles) was added along with PBS.

### Characterization of MNNPs

The synthesized MNNPs were characterized in detail by using, FTIR, FE-SEM, TEM and DLS.

#### Scanning electron microscopy

The surface morphology and shape of synthesized NPs was achieved by Field Emission Scanning Electron Microscopy (FE-SEM) (MIRA3 TESCAN, Czech Republic). FE-SEM system at an accelerating voltage of 8 kV was used to take the images of synthesized MNIONPs. For taking the FE-SEM images, the synthesized NPs were mixed with acetone and allowed to dry on a glass slide in order to get a thin layer for the analysis.

#### Transmission electron microscopy (TEM)

TEM was performed on JEOL JEM-1010 TEM (Tokyo, Japan) at an accelerating voltage of between 10 and 80 kV. The samples for TEM were prepared by placing a drop of the prepared particle suspension on a copper grid containing carbon film (VWR, 100,503–154) and dried overnight in desiccator at 25 °C.

#### Measurements of particle size distribution and zeta potential

The particle size, polydispersity index (PDI) and surface charge (zeta potential, mV) was assessed using zeta potential measurements collected via dynamic light scattering (DLS) Zetasizer Nano ZS (Malvern Instruments Ltd., Malvern, UK) equipped with a helium– neon laser beam at a fixed scattering angle of 90 and a wavelength of 633 nm. For this purpose, NPs were dissolved in deionized water at a concentration of 0.5 mg/mL followed by 10 min sonication at 25 ℃.

#### FTIR spectroscopy

Fourier transform infrared (FTIR) (Shimadzu 8400S, Japan) spectroscopy was used to examine the surface functional groups of the RSV loaded MNNPs in the infrared range 400 to 4000 cm^1^. Vibrating sample magnetometer (VSM) (MDK, Iran) was used to assess the magnetization value of MNNPs.

### Drug loading (DL) and encapsulation efficiency (EE) of MNPs

EE % is defined as the portion of the applied drug which is entrapped by the niosomes. The EE % of RSV in the MNIONPs was determined using an indirect technique. After freshly prepared MNIONPs were formed, the dispersion was centrifuged at 13,000 rpm, 30 min. The concentration of RSV in the supernatant was determined to calculate the EE using Eq. 1. The DL % was calculated through Eq. 1. All the measurements were performed in triplicate.

The direct method was used to determine the drug loading percentage of RSV in the MNIONPs. The measured lyophilized powder was dissolved in the 1 mL of methanol. The solution was then completed to 3 mL with acetonitrile and subjected to ultrasonic bath for 45 min. The RSV was extracted into acetonitrile. Next, the solution was filtered through a membrane filter. (Millipore, HA, 0.45 µm) and measured in UV spectrophotometer at 324.93 nm (n = 3). The drug loading values of MNIONPs were calculated using Eq. 2.

1 $${\text{EE}}=\frac{\mathrm{Weight \,of \,drug \,in \,MNPs}}{\mathrm{total \,Weight \,drug}} \times 100\mathrm{\%}$$

2 $${\text{DL}}=\frac{\mathrm{weight \,of \,drug \,in \,MNPs}}{\mathrm{total \,NPs \,weight}}\times 100\mathrm{\%}$$

### In vitro RSV release assay

The dialysis technique was used to assess the release behavior of RSV from RSV-MNIONPs. For in vitro drugs discharge analyze, 10–15 mg RSV loaded MNPs were dispersed in PBS (5 ml, pH = 7.4 and pH = 5.8) and transferred to dialysis bag (MW = 12 kDa) located in 20 ml of PBS with stirring at 120 rpm at 37 ℃. After that the sample was periodically removed from the incubation medium and rapidly substituted with an equal quantity of fresh PBS. A UV–Vis spectrometer was used to determine the release of RSV at 324.93 nm. Statistical analysis of the data was done using analysis of variance (single factor). Difference was considered significant when *P* < 0.05.

### Cell culture

The pancreatic Capan-1 cell line was purchased from National Cell Bank of Iran (Pasteur Institute of Iran, Tehran). The cells were cultured in RPMI 1640 with 100 units/mL penicillin–streptomycin (P/S) and 10% fetal bovine serum (FBS) at 37 ℃ and 5% CO_2_, followed by subculture every two to 3 days.

### RSV-MNIONPs cellular uptake experiments

The cellular internalization of RSV-MNIONPs was studied using the Capan-1 cell line and analyzed by a flow cytometry device. For flow cytometric analysis, Capan-1 cells were seeded in a 6-well plate at a cell density of 3 × 10^5^ cells per well in RPMI 1640 medium and incubated for 24 h with Blank, RSV and RSV-MNIONPs at 37 ℃. Then, the cells were rinsed 3 times with PBS, digested with trypsin and collected by centrifugation. Next, the cells were incubated with the FITC that was dissolved in 250 μL of methanol and mixed with 1 mL of niosomal complexes for 3 h at 37 ℃. The extents of intracellular uptake of RSV-MNIONPs by the cells were monitored by flow cytometry (FACScan, Becton Dickinson).

### Cell viability assay

A MTT assay was carried out to evaluate the cytotoxicity of free RSV and RSV-MNIONPs after 24,48 and 72 h of exposure to the medication in the presence of an external magnetic field [24]. In brief, 2 × 10^5^ Capan-1 cells and peripheral blood mononuclear cells (PBMCs) cell were seeded on a 96-well plate and were treated with various concentrations (0, 1, 5, 30, and 50 μM) of RSV and RSV loaded MNPs for 24,48 and 72 h. Next, after removing the RPMI-1640 medium, an amount of 200 µL MTT solution was added to each well, with subsequent incubation for 4 h. Following this, 200 μL of DMSO substituted the MTT mixture and it was shaken in a shaker for 20 min. After shaking for 20 min, the absorbance at 490 nm was determined using an ELISA plate reader (Bio-Tek Instruments), taking 630 nm as its reference wavelength. All the experiments were done in triplicate. The cells treated with a culture medium were used as a control. The cytotoxicity of RSV and RSV-MNIONPs was expressed as IC_50_. This is defined as the drug concentration needed to inhibit cell growth by 50% comparative to the control. These values were determined by nonlinear regression analysis of the response curves via GraphPad Prism 9. The cell growth inhibition on Capan-1 cells was determined as follows:

$$\,{\text{Cell growth inhibition}} = {{\left( {{\text{OD }}570{\text{ control}} - {\text{OD }}570{\text{ sample}}} \right)} \mathord{\left/ {\vphantom {{\left( {{\text{OD }}570{\text{ control}} - {\text{OD }}570{\text{ sample}}} \right)} {\left( {{\text{OD }}570{\text{ control }}} \right)}}} \right. \kern-0pt} {\left( {{\text{OD }}570{\text{ control }}} \right)}} \times 100$$

### Cell cycle analysis

About 5 × 10^5^ Capan-1 cells were seeded at per well of 6-well plates and incubated for 24 h. Then cells were treated with free RSV and RSV-MNIONPs. Following 48 h of drug exposure, the cell debris and aggregates were collected and washed three times with PBS (pH = 7.4) before being fixed with 70% ethanol and stored at –20 ℃ for 48 h. The cells were taken, re-suspended in PBS (pH = 7.4), and stained with a solution which contains RNase A and PI then the cells incubated for 30 min at 37 ℃. The fluorescence was read using the flow cytometer.

### PI/Annexin V staining

Flow cytometry was assessed to analyzed apoptosis induction. Briefly, Capan-1 cells were plated in 6-well plates (∼2 × 10^5^ cells/well) and exposed with free RSV and RSV-MNIONPs for 48 h. Cells were trypsinized, and then washed with PBS (pH = 7.4). Falcons were centrifuged for 5 min and 1000 rpm, followed by supernatant discarding and 1 mL of PBS was added. Centrifuging was repeated, and 100 μL binding buffer, 5 μL annexin V and 5 μL propidium iodide (PI) was added to each sample and left in the dark for 20 min. Finally, 100 μL of binding buffer was added, and reading was performed using a flow cytometry device (Thermo Fisher Scientific Co, USA) in the darkness. To determine the percentage of apoptotic cells Annexin V + /PI − cells were designated as early apoptotic cells, whereas Annexin V + /PI + cells were identified as late apoptotic cells.

### RNA extraction, cDNA synthesis and real-time PCR

Real-time PCR test was applied to assess the transcription mRNA of the FAS, BAX, BCL- 2, P 53, cycline D and hTERT genes. The first 2 × 10^6^ Capan-1 cells were seeded in 6-well plates and incubated at 37 ℃ with 5% CO_2_. After 48 h cells were exposed separately to free RSV and RSV-MNIONPs at their IC_50_ concentration, total mRNA was extracted by Trizol (Sigma, Germany) reagent based on the instrument. Then, total RNA was reverse-transcribed to complementary DNA (cDNA) using the SinaClon First Strand cDNA synthesis kit (SinaClone, Theran, Iran). According to the manufacturer’s protocols, the genes expression levels were then evaluated using the qPCR technique employing the Hot Taq EvaGreen qPCR. The qPCR included a primary activation phase for 5 min at 94 ℃, followed by 35 cycles for 30 s at 94 ℃, annealing for 30 s at 60 ℃, extension for 30 s at 72 ℃, and a final extension for 7 min at 72 ℃. The primer-blast on the NCBI website was used to blast the exact primers that were applied for real-time PCR. Finally, the housekeeping gene (GAPDH) was used to normalize the relative expression levels of the stated genes. Furthermore, the 2^−ΔΔCT^ equation was used to determine these levels.

### Statistical assessment

All results are presented as the mean ± SD (*p < 0.05, ** p < 0.01, and *** p < 0.001). For in vitro experiments, student’s T‐test was employed with GraphPad Prism 6.0 software, and Real-time PCR data were analyzed statistically using REST 2009 software.

## Results

### Characterization of synthesized RSV-MNIONPs

#### Measurements of particle size distribution and zeta potential

**Fig. 1:**
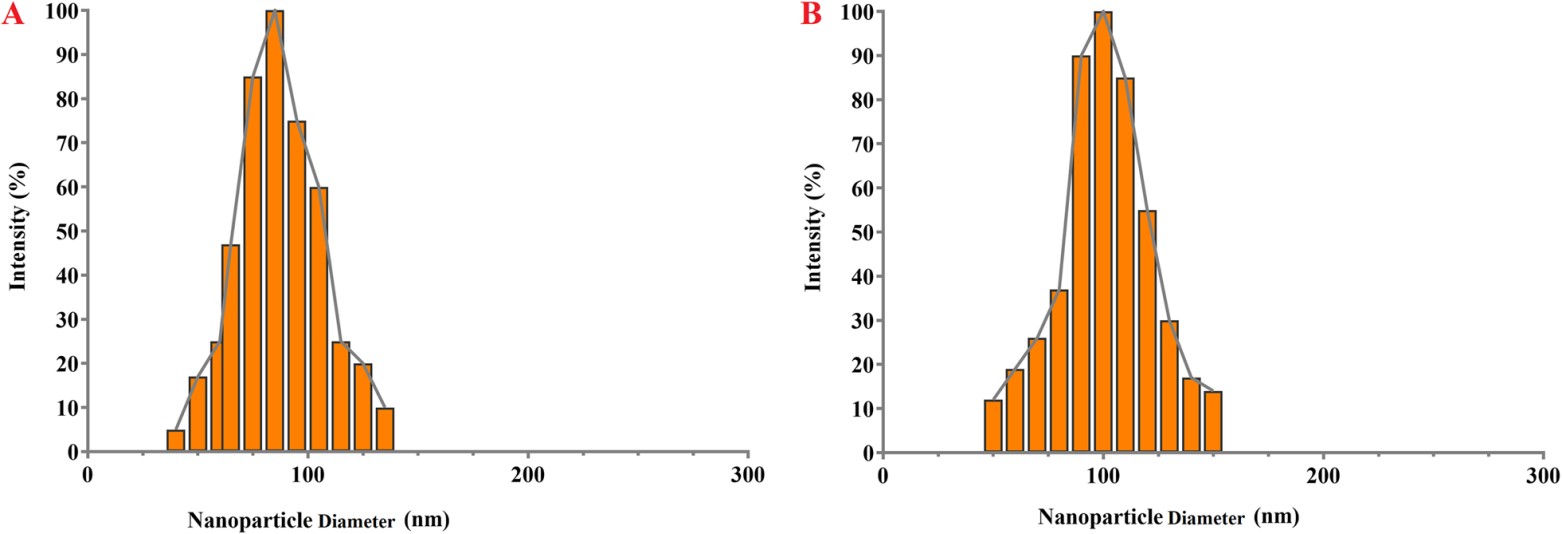
The DLS analysis on mean diameter of MNIO NPs and RSV-MNIO NPs

RSV-loaded MNIONPs prepared using the thin film hydration technique. The surface charge and size of the prepared RSV-MNIONPs were analyzed using zeta potential analysis and DLS technique. The particle size analysis via DLS showed the uniform dispersion of particles with an average size of 70 ± 8.3 nm, a polydispersity index of 0.127 ± 0.034 and zeta potential of − 12.06 ± 3.4 mV for MNIONPs. Moreover, it has been showed that RSV-MNIONPs displayed an median diameter of 105 ± 3.6 nm, a polydispersity index of 0.243 ± 0.054 and zeta potential of − 9.03 ± 1.5 mV. (Fig. 1 and Table 1).

**Table 1:** Physicochemical data parameters of MNIONPs and RSV-MNIONPs. Results means ± SD (n = 3)

Groups	Particle size (nm)** ± **SD	Polydispersity** ± **SD	Zeta potential (mV)
MNIONPs	70 ± 8.3	0.127 ± 0.034	− 12.06 ± 3.4
RSV-MNIONPs	105 ± 3.6	0.243 ± 0.054	− 9.03 ± 1.5

#### Microscopic analysis of niosomes

**Fig. 2:**
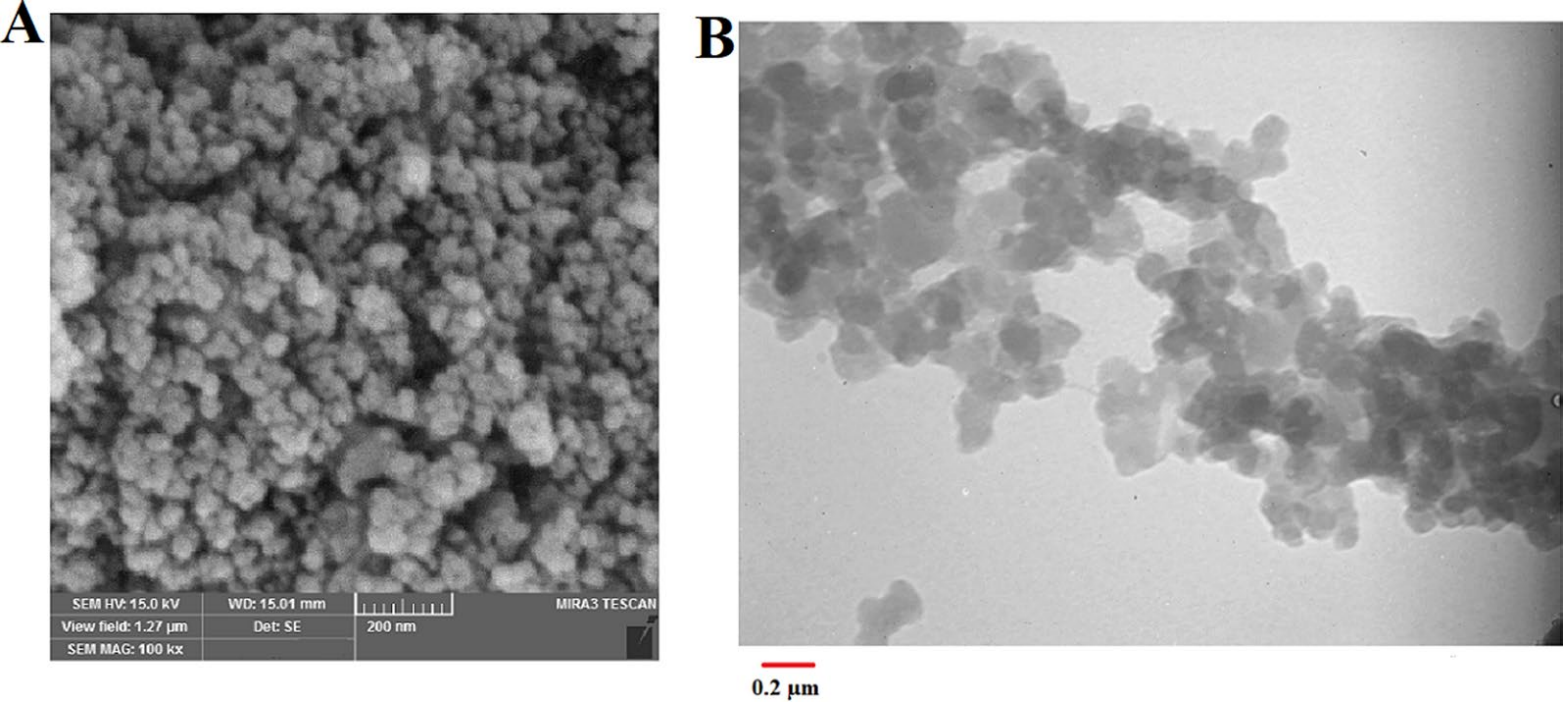
SEM (**A**) and TEM (**B**) analysis of the MNIO NPs and RSV-MNIO NPs

The evolution of size, shape and surface morphology of prepared NPs was studied with SEM and TEM techniques. According to the SEM image, shapes and distributions of MNNPs were homogeneous spherical with a size of 100 nm (Fig. 2A). The size acquired from TEM was almost comparable with the hydrodynamic diameter determined through particle size analyzer (Fig. 2B).

#### FTIR analysis

**Fig. 3:**
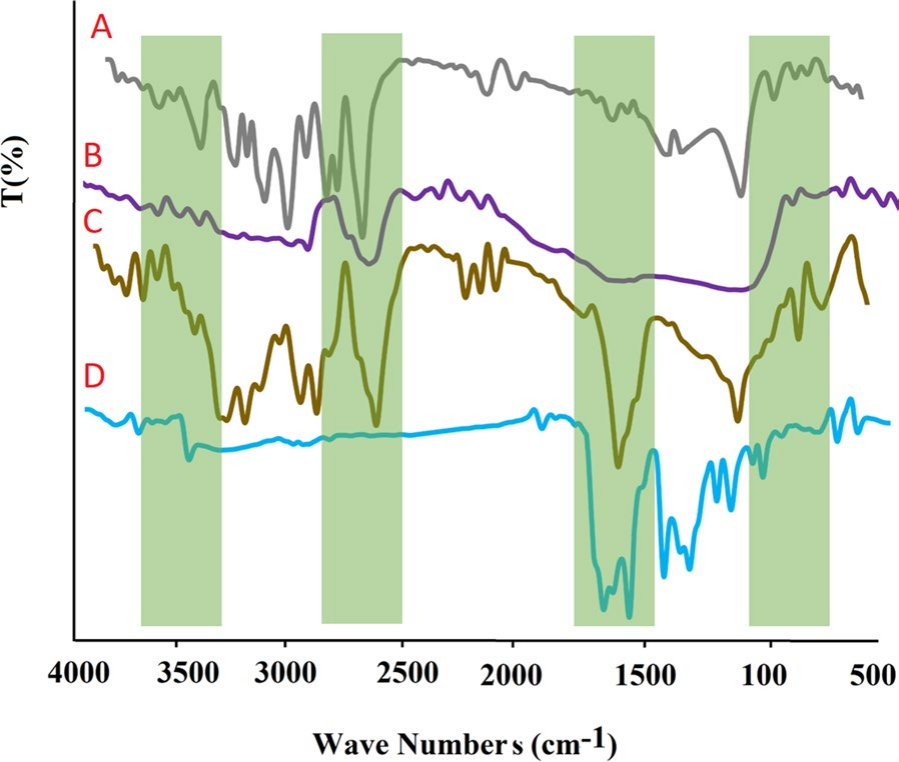
FTIR spectrum for **A** MNPs, **B** NIOs, **C** RSV, and **D** RSV-MNIO NPs

FT-IR analysis was done for MNPs, NIOs, MNNPs, and RSV loaded MNNPs were monitored, which indicated that RSV and MNPs were loaded in NIOs (Fig. 3). The spectrum of free RSV displays broad peaks at 3200 cm^−1^, which are related to O–H bonds of drug. A strong peak in the range of 1435 cm^−1^ indicates the O–C = O bond of NIOs. The peak at 1112 cm^−1^ signify the C–C stretching vibrations of RSV loaded MNNPs. The broad bands at 3408 cm^−1^ and 3456 cm^−1^ is because of the presence of OH stretching of RSV loaded MNNPs and RSV. In the case of RSV loaded MNNPs, the presence of all characteristic peaks of NIOs and RSV showed the successful integration and loading of RSV and NIOs into RSV loaded MNNPs.

**Fig. 4:**
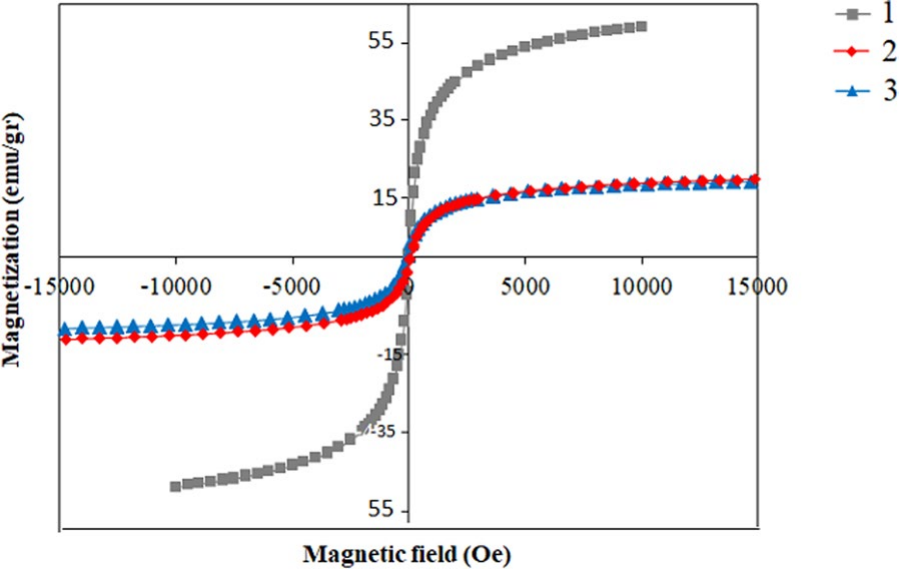
The magnetic behavior of magnetic nanoparticles. (1. Fe3O4 NPs, 2. MNIO NPs 3. RSV-MNIO NPs

The magnetic activities of RSV loaded MNNPs were demonstrated using VSM at room temperature (Fig. 4). This difference approves that the loading of MNPs and RSV in NIOs is done correctly, according to preceding reports distributed in this area. As you seen in Fig. 4, the results show that there is none hysteresis curve signifying the superparamagnetic behavior of the prepared particles.

### DL, EE efficiency and in vitro evaluation drug release kinetics

Drug loading efficiency is defined as the ratio of the amount of drug in the NPs to the total amount of drug used in the formulation of the NPs. These days, due to the low efficiency drug loading of many drug delivery methods, it seems necessary to develop new drug delivery systems.

Both the EE and DL analyses presented that RSV were successfully loaded into MNIONPs. It was found that RSV loaded into MNIONPs were exhibited the encapsulation efficiencies of 90.38 ± 0.1% with loading capacities of 13.2 ± 4.3%.

**Fig. 5:**
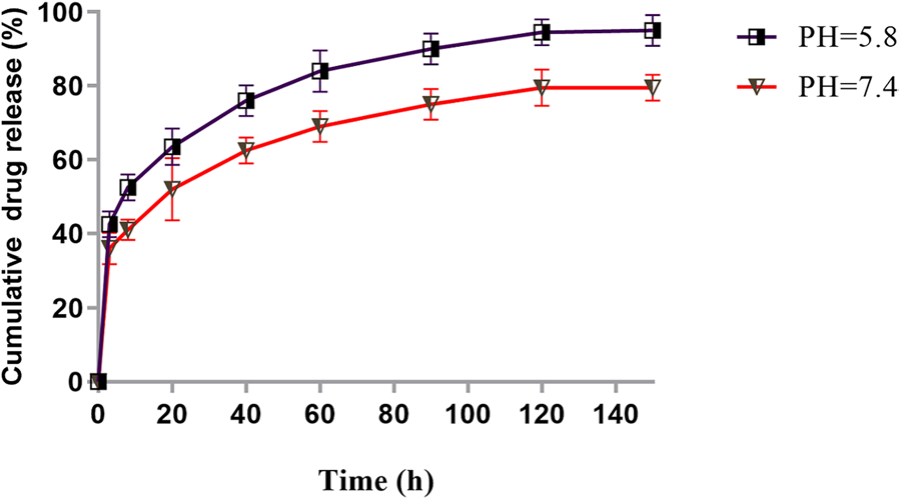
In vitro release kinetics of RSV form RSV-MNIO NPs at pH 5.8 and pH 7.4. The data are presented as mean ± SD (n = 3)

To study the role of biochemical and chemical factors on the RSV release from MNIONPs, the work was used on the RSV-MNIONPs in neutral (physiological pH of blood stream, pH = 7.4) and acidified pH (mimicking microenvironment in endosomes and lysosomes, pH = 5.8) PBS solution. The cumulative proportion of RSV released from RSV-MNIONPs during various time periods (0, 20, 40, 60, 80,100,120 and 140 h) was displayed in Fig. 5. Discharge of RSV from RSV-MNIONPs were happened in two phases. Initially, a sustained release was done for 4 h at burst drug release rate, allowing for the release of 40% of the entrapped RSV. After that, a prolonged phase was done for 140 h at a decreased and light release rate, and the maximum discharge of RSV from MNIONPs reached 85% of the total entrapped drug which was shown that continuity of RSV in MNIONPs at pH 7.4 is acceptable. This two-phase discharge pattern is in consistence with preceding observations and appears to be a property of bilayer vesicles in general.

### Cell cytotoxicity of MNNPs

**Fig. 6:**
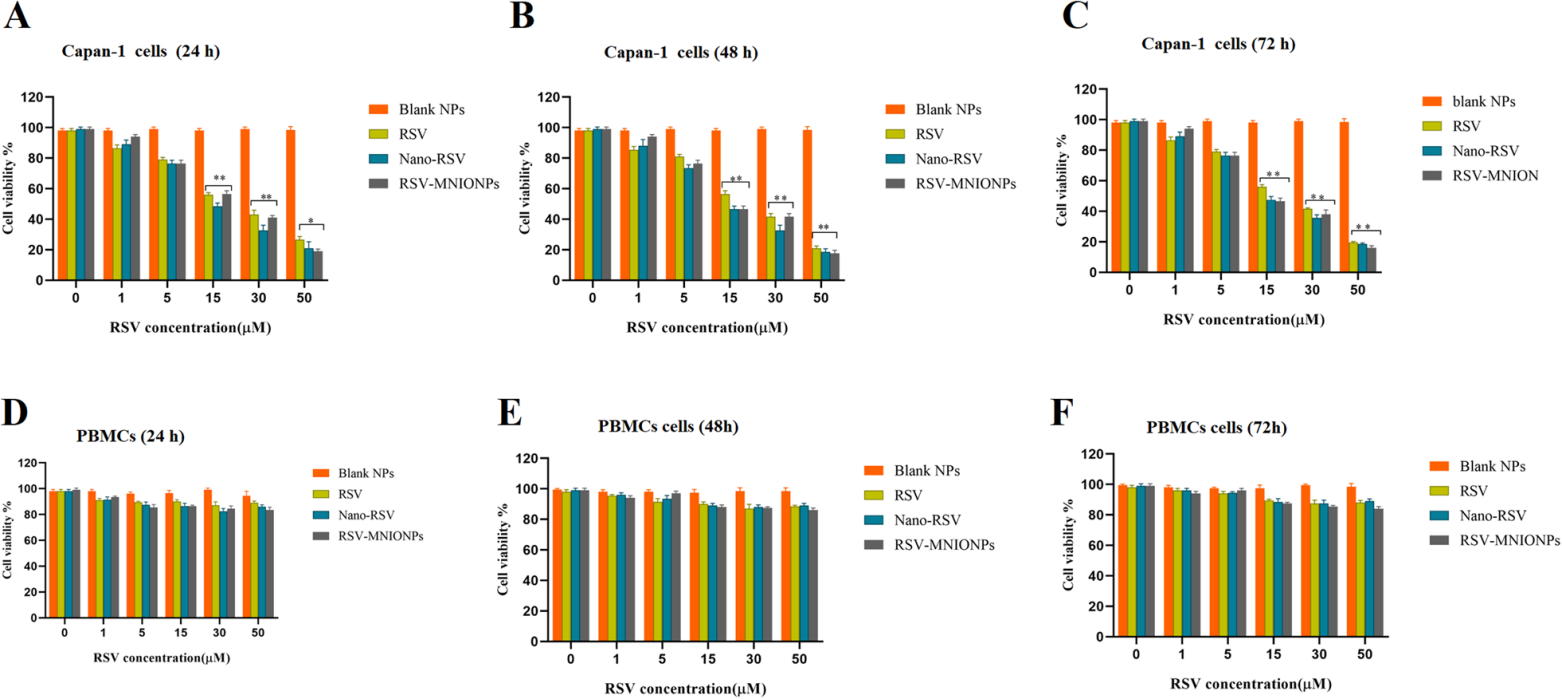
In vitro cytotoxicity of Blank NPs, RSV, MNIO NPs and RSV-MNIO NPs in Capan-1 cell and PBMCs cell in the presence of a magnetic field: Cell viability of Capan-1 cell and PBMCs cell after treatment Blank NPs, RSV, RSV-NIO NPs and RSV-MNIO NPs for **A** and **D** 24, **B** and **E** 48 and 72 h (**C** and **F**). Data represents in the form of mean (n = 3) ± SD with p value < 0.0001 (***), < 0.001 (**) and < 0.01 (*)

In the cell cytotoxicity assessment by the MTT method, Capan-1 and PBMCs cells were treated with free RSV and RSV-MNIONPs for 24, 48 and 72 h at various concentrations (0, 1, 5, 15, 30 and 50 μg/mL) in the presence of a magnetic field (Fig. 6). A dose and time-response chart was planned based on the viability rate of cells 24, 48 h and 72 h after treatment with different doses of free RSV and RSV-MNIONPs (Fig. 6). The IC_50_ values for these three intervals were determined. RSV-MNIONPs displayed powerful inhibitory activity against Capan-1 cells with an IC_50_ noticeably lower than that of free RSV. In keeping with the results, RSV-MNIONPs has cytotoxic effects of 25, 18 and 12 μg/mL in 24, 48 and 72 h, respectively (Fig. 6A–C). In contrast, free RSV has a lower cytotoxic effect than RSV-MNIONPs and needs higher concentrations to affect cells. As shown in Fig. 6, the IC_50_ values of free RSV were 30, 20 and 15 μg/mL at 24, 48 and 72 h, respectively. To verify whether RSV and RSV-MNIONPs at different concentration are toxic to normal cells, PBMC cells were exposed with RSV and RSV-MNIONPs (0, 1, 5, 15, 30 and 50 μg/mL) for 24, 48, and 72 h. Both RSV and RSV-MNIONPs showed a slight toxic influence on PBMC cells viability (Fig. 6D–F).

### Cellular uptake analysis on capan-1 cell line

**Fig. 7:**
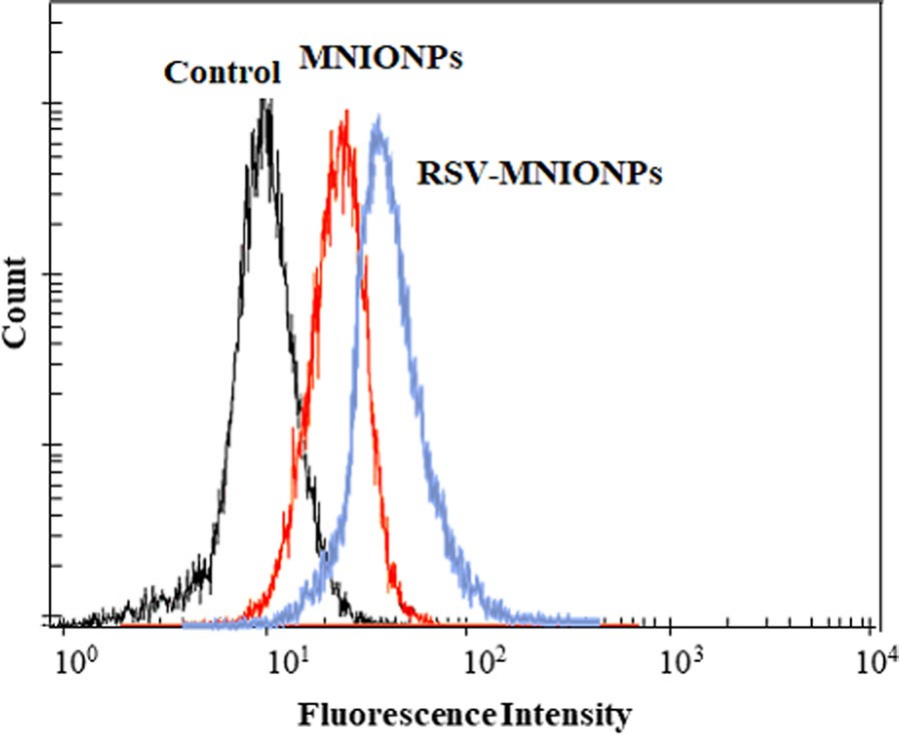
Rhodamine b label RSV-MNIO NPs and MNIO NPs uptake by Chapan-1 cell after 3 h by FACS

Figure. 7 displayed the cellular uptake level of RSV-MNIONPs by the Capan-1cell line was 99% compared to the control group. These results showed that the MNNPs could increase cellular uptake meaningfully and prompt apoptosis in the Capan-1 cell line after treatment, and are consistent with preceding studies that used flow cytometry to examine apoptosis and MNP uptake in cells [29, 30].

### Cell cycle arrest analysis

**Fig. 8:**
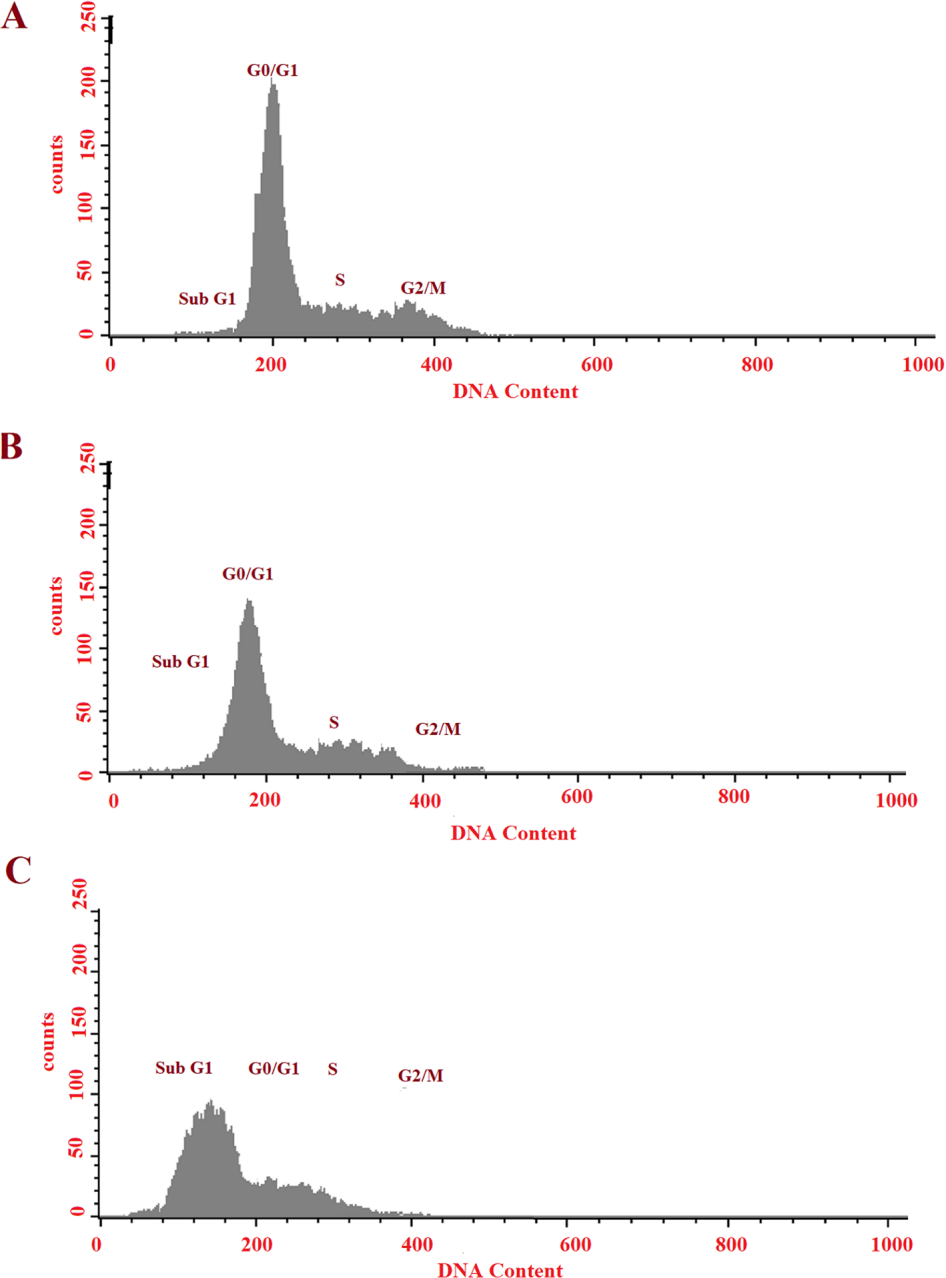
Suppression effects of free RSV, RSV- NIOs and RSV-MNIONPs on cycle of Capan-1 cell. Schematic figure of RSV, RSV- NIOs and RSV-MNIONPs on cell cycle arrest of Capan-1 cell line. **A** Control, **B** free RSV, **C** RSV-NIOs, and **C** RSV-MNIONPs

In current work, we evaluated the effect of free RSV, Nano-RSV and RSV-MNIONPs on cell cycle of Capan-1cell line. As displayed in Fig. 8, cell populations were 24.4%, 65.3%, 3.73%, and 7.24% at the Sub-G0/G1, G0/G1, S, and G2 phases after 24 h of treatment with RSV-MNIONPs (Fig. 8). The corresponding percentages for free RSV were 43.4%, 40.4%, 10.53%, and 6.11%, respectively (Fig. 8B). Compared with control, both free and nano-formulate form of RSV showed a greater shift toward G0/G1. The shift in cell population distribution towards the G0/G1 phase suggests the initiation of DNA damage responses. While cells exposed to RSV-MNIONPs showed a similar pattern to free RSV, RSV-MNIONPs accelerated cell cycle arrest at G0/G1, suggesting early onset of apoptosis. Our results are in agreement with the preceding reports stated that RSV could suppress the cell cycle at phase G1 and nano capsulation of these drugs had a higher effect. This suggests that the noisome base of the combination of RSV can suppress the development of Capan-1cell line and improve the survival rate.

### PI/Annexin V staining

**Fig. 9:**
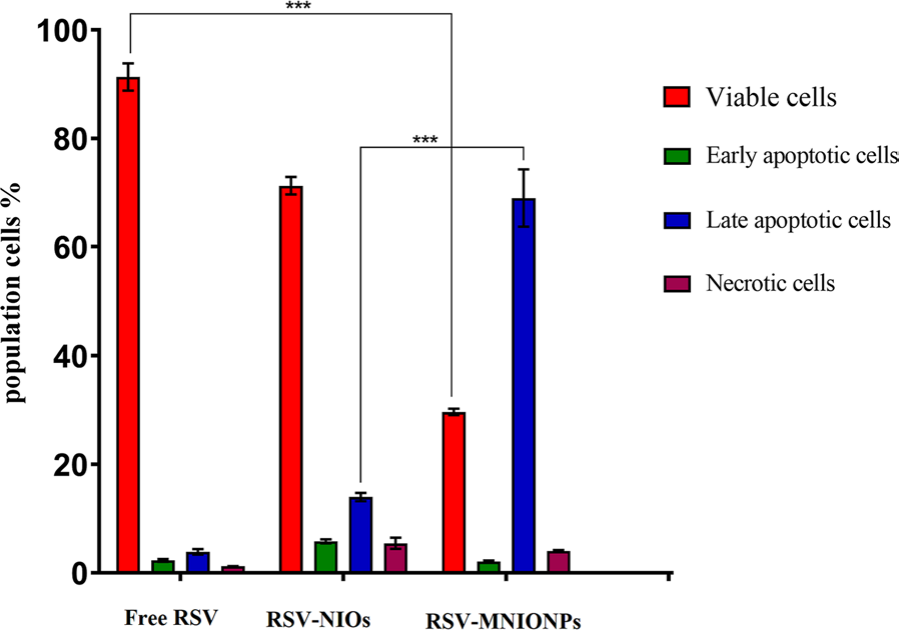
Apoptosis analysis on Capan-1 cell line under treatment of free RSV, RSV- NIOs and RSV-MNIONPs. Capan-1 cells treated with pure RSV drug and niosomal complexes were evaluated after 48 h by FITC-labeled annexin V/PI flow cytometry. The obtained results confirm that NIOs loaded with the RSV significantly increase the apoptosis of Capan-1 cells, which emphasizes the increase in the absorption of drugs from NIOs by the cells. This result is consistent with the MTT results

To quantify the proportion of dead and apoptotic Capan-1cell line following treatment with free RSV and RSV-MNIONPs, PI/Annexin V staining was utilized (Fig. 9). As presented in Fig. 7, in comparison to control, free RSV and RSV-MNIONPs caused apoptosis under experimental conditions. Our results showed that after 24 h of treatment with IC_50_ concentrations of RSV-MNIONPs, most of the cells are in the late stage of apoptosis (68.3%). These results proposed that free RSV and RSV-MNIONPs could prompt apoptosis in Capan-1cell line. Nevertheless, it is clear that free RSV needs a higher concentration to prompt cytotoxicity in comparison to RSV-MNIONPs. Generally, our finding presented that there is noteworthy difference between the effects of these free RSV and RSV-MNIONPs in IC_50_ concentration. Altogether, the results came from Annexin V- FITC/PI showed that RSV-MNIONPs effectively induced apoptosis in Capan-1cell.

### The analysis of gene expression

**Fig. 10:**
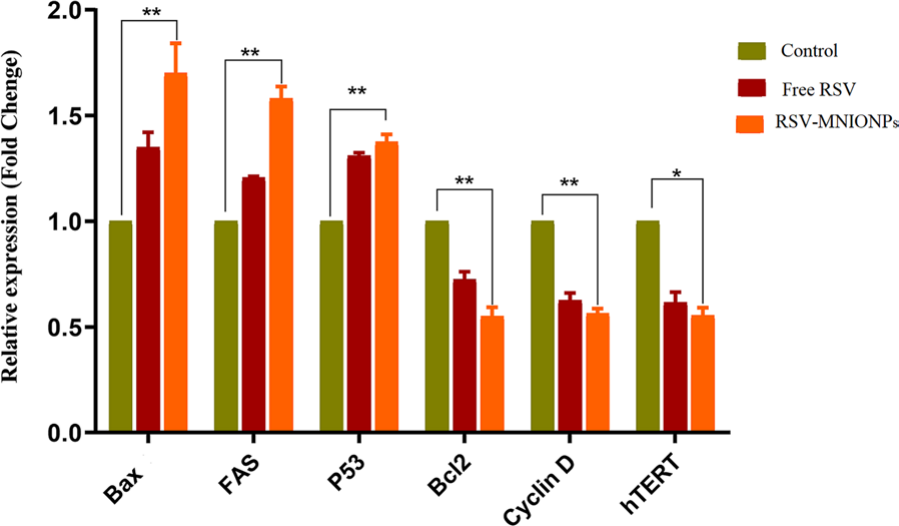
Inhibitory effects of free RSV, RSV- NIOs and RSV-MNIONPs on expression levels of P53, FAS, Cyclin D, hTERT, BAX and Bcl-2 in Capan-1 cancer cells. *P < 0.05 and **P < 0.01 are the statistical differences between the combination form and individual drugs. Data represented are from three independent experiments

To study the anti-tumor effects of encapsulation and free form of RSV in Capan-1cell line, the expression mRNA level of apoptotic related genes including BAX and BCL-2 m P53, cycline D and Fas genes involved in cancer cells were measured by RT-PCR. Our results showed that RSV in nano-capsulated and free form down-regulated the expression of cycline D, hTERT and BCL-2 while upregulated the expression of BAX, Fas and P53 (Fig. 10). This is demonstrated that simultaneous use of phytochemical and magnetic NPs may increase the anti-tumor effects and have a synergistic apoptosis effect on cancer cells. This procedure could be useful to raise the therapeutic benefit of individual treatment, mainly in the eradication of hindrances such as low drug efficacy and tumor resistance. Our results are in consistent with a current study that has revealed that RSV alone and in nanoformulations form could downregulate the expression of hTERT gene.

## Discussion

NIOs are vesicular drug delivery systems composed of nonionic surfactants, cholesterol, and sometimes other additives. They are similar to liposomes but are made from nonionic surfactants rather than phospholipids. The use of nonionic surfactants provides NIOs with numerous advantages, such as superior physicochemical stability and lower production costs in comparison with liposomes [39].

Commonly, NPs size is one of the important parameters about on the release of the drug from the NPs, physiochemical stability, biological distribution and cellular uptake. The different sizes have been described by preceding study, and it appears that other physical aspect s of magnetic NPs like surface charge as well as some structures of chemical compounds and synthesis techniques are accountable for the size of magnetic NPs [40]. Numerous reports have revealed that the surface charge of NPs plays a crucial role in their interaction with cells and subsequent internalization. Positively charged NPs often exhibit higher cellular internalization compared to neutral or negatively charged counterparts, and this phenomenon is attributed to several factors including enhanced cell membrane penetration, cellular uptake pathways and influence on intracellular trafficking [41, 42]. In current work, the surface charge of the prepared RSV-MNIONPs was determined using the DLS technique as the zeta potential was − 12.06 ± 3.4 and polydispersity index (PDI) was 0.127 ± 0.034. The zeta potential outcome shows that the surface charges of prepared RSV-MNIONPs are negative.

A VSM is a scientific instrument used to measure the magnetic properties of materials. It is particularly useful for studying the magnetization of solid materials as a function of an applied magnetic field [43, 44]. It’s important to note that achieving these benefits requires a careful balance in the design and characteristics of the MNPs, taking into account factors such as size, composition, and surface properties. Scientists in the field of nanomedicine endlessly discover approaches to improve MNPs for numerous biomedical uses, such as magnetic hyperthermia for treatment of cancer [45].

Drug release from vesicular systems, including liposomes and NIOs, often occurs through passive processes such as diffusion or degradation of the vesicle membrane. The relationship between vesicle size, drug entrapment efficiency, and release rate in vesicular drug delivery systems is complex and can depend on various factors [46]. Unquestionably, attaining prolonged drug retaining and controlled, delayed release are serious purposes when designing an ideal drug delivery system. Our results show that the controlled release rates of RSV from MNNIOs are meaningfully slower than free form solution rate.

Jin et al. confirmed that decreasing the pH is a hopeful approach to improvement the DL efficiency up to around 70% in the MNPs formation whereas this efficacy minus pH alteration is between 10 and 15% [47]. In a study by Barani et al. confirmed that the drug's discharge pattern from MNPs presented a first rapid release, followed by a sustained and longer-lasting release in acidic conditions [25, 26]. In another study, the efficacy of doxorubicin-loaded MNNPs loading for the treatment of cancer was analyzed and revealed that the utilization of MNNPs can improve the release rate of formulations [27].

Hajinezhad and et al. designed a novel encapsulate ifosfamide (IFO) niosomal nanometric size range with a high drug-loading capacity and evaluate the anticancer effectiveness of the encapsulate IFO. They result showed that niosomal IFO had desirable anticancer activity against breast cancer (MCF7) and neuroblastoma (SH-SY5Y) cells with a high potential in the controlled release of IFO [28].

Our results showed that free RSV and RSV-MNIONPs in different concentrations have less toxic effects on PBMCs normal cells compared to Capan-1 cancer cells. The impact of MNPs on normal cells versus cancer cells can vary based on factors such as NPs properties, concentration, exposure time, and the specific characteristics of the cell types involved. Both normal and cancer cells may internalize RSV-MNIONPs, but the extent of uptake can depend on factors such as particle size, surface charge, and targeting ligands. Cancer cells often exhibit higher rates of endocytosis compared to normal cells, potentially leading to increased internalization of magnetic nanoparticles [48].

In a study, chrysin nanoformulated in niosomes presented antitumor effects on human SKOV3 ovarian cancer cell line by decreasing growth of cancer cell and stimulation of antitoxic impacts [49]. Anticancer efficacy of RSV-loaded in liposomes evaluated and showed that their have anti-proliferative effect on U-87MG cells by inhibiting cell growth and inducing apoptosis in nude mice [50]. RSV-loaded liposomes have shown to be effective anti-proliferative agents in brain cancer cells such as U-87MG cells through inhibiting cell growth and inducing apoptosis in nude mice [50]. Our results confirm the previous studies reported that the cytotoxicity of the drug-loaded MNNPs follows dose-dependent and time-dependent trends [27].

Effectively internalized by cancer cell is an important requirement for any nanomaterials used for theranostics such as photodynamic therapy. Thus, the cellular uptake of RSV-MNIONPs was carefully studied through flow cytometry [30]. Our results showed that RSV-MNIONPs compared with free RSV and control internalized more into the cell .This results indicated that MNPs in the presence of magnetic field cause more drug internalization.

The cell cycle is highly regulated by several pathways confirming organized and coordinated cell division. Cell cycle arrest is the primary mechanism by which many anticancer drugs inhibit tumor cell proliferation [31]. Understanding the mechanisms of the endless cell cycle in tumor cells could develop the new combination therapy [32, 33]. Recent studies have shown that RSV could induce cell cycle arrest in most cancer cells [32]. In a study by Karthikeyan et al. RSV-loaded gelatin nanoparticles (GNPs) and shown that combination of RSV with GNPs altered expression of apoptosis genes and prompted cell arrest in the G0/G1 phase of cell cycle in NCI-H460 cells. Our results revealed that both free and nano-formulate form of RSV in comparison with control showed a greater shift toward G0/G1. This outcome is well supported by our apoptosis analyses and it is in agreement with the results of preceding studies.

The effectiveness of antitumor agents is commonly defined through the apoptosis and cell survival. Apoptosis is a natural process that acts a critical role in preserving the balance of cell growth and death in dividing cells [51]. In current study, the apoptosis rate of Capan-1 cells was calculated after treatment with various examples. These outcomes are according to previous investigation, which revealed that the internalization and following intracellular release of the antitumor agent from NPs is accountable for the cytotoxic effect of NPs [52]. It sounds like, the proposed formulation has the potential to improve drug bioavailability. This improvement could cause the induction of apoptosis in cancer cells through mechanisms such as DNA damage or the oxidation of lipids, proteins, and enzymes [53].

BAX and BCL-2, belong to BCL-2 family that controls the apoptosis procedure through the mitochondrial path [34]. A current report has revealed that normal expression of BAX in tumor cells was related with better consequences, while expression of BCL-2 in cancer cells affected drug resistance. Consequently, it is predicted that changing the expression of these genes will cause apoptosis and inhibit the cancer cells growth [35].

Unusual expression of telomerase is found in numerous tumors that have an critical act in the propagation and immortality of cancer cells, which is a characteristic of cancer cells [36]. This property of telomerase becomes a possible target in drug delivery and treatment of cancer. It is believed that regulation of hTERT's transcription, which triggers telomerase in a kind of malignancies, is critical [37]. Moreover, it has been confirmed that the hindering of hTERT accelerates the stimulation of apoptosis in tumor cells. Therefore, hopeful procedure in treating of pancratic cancer may be suppress gene expression as well as trigger apoptosis [38].

Meanwhile no special management/storing provisions are required for NIOs, their marketable exploitation would be at ease. Furthermore, presence surfactant in structure, they have acquired a capability to thorough’s phagocytic protection mechanism and doing as covertness drug transporters making their efficient circulation time longer than the drug given Inc usual formulations. In summary, magnetic niosomal NPs have the potential to revolutionize drug delivery, imaging, and therapy, with ongoing research focused on addressing challenges and unlocking their full clinical potential. The integration of nanotechnology with magnetic guidance opens up new opportunities for more efficient and targeted medical interventions.

## Conclusion

In summary this study has confirmed the significance of using RSV-MNIONPs compared to nanformolated form of RSV treatment in the presence of a magnetic field. We developed a nanocarrier system based MNIONPs through thin-film hydration method and loaded the antitumor RSV. The prepared NPs characterized by FT-IR, DLS SEM, TEM and VSM to prove preparation of MNIONPs. NIOs delivery system reveals the high EE% with a contentious release profile. Cytotoxicity assay for RSV-MNIO NPs against Chapan-1 cancer cell lines showed that the IC_50_ of free form of drugs were significantly higher than the drugs loaded on magnetic niosomes in the presence of a magnetic field. This results demonstrated that a relatively prolonged release of RSV through encapsulation in MNIONPs could decrease the dose needed to kill cancer cells. Moreover, RSV-MNIO NPs caused more apoptosis against Chapan-1 cells than free RSV, as confirmed by cytotoxicity and genotoxicity studies. Results showed that RSV-MNIO NPs may kill Chapan-1 more rapidly than either free RSV therapy, proposing that this approach may reduce cytotoxicity and adverse effects on patients.

## Data Availability

Not applicable.
